# Non-specific psychological distress, smoking status and smoking cessation: United States National Health Interview Survey 2005

**DOI:** 10.1186/1471-2458-11-256

**Published:** 2011-04-22

**Authors:** David Lawrence, Francis Mitrou, Stephen R Zubrick

**Affiliations:** 1 1Telethon Institute for Child Health Research, Centre for Child Health Research, The University of Western Australia, P.O. Box 855, West Perth, WA. 6872. Australia

## Abstract

**Background:**

It is well established that smoking rates in people with common mental disorders such as anxiety or depressive disorders are much higher than in people without mental disorders. It is less clear whether people with these mental disorders want to quit smoking, attempt to quit smoking or successfully quit smoking at the same rate as people without such disorders.

**Methods:**

We used data from the 2005 Cancer Control Supplement to the United States National Health Interview Survey to explore the relationship between psychological distress as measured using the K6 scale and smoking cessation, by comparing current smokers who had tried unsuccessfully to quit in the previous 12 months to people able to quit for at least 7 to 24 months prior to the survey. We also used data from the 2007 Australian National Survey of Mental Health and Wellbeing to examine the relationship between psychological distress (K6) scores and duration of mental illness.

**Results:**

The majority of people with high K6 psychological distress scores also meet diagnostic criteria for mental disorders, and over 90% of these people had first onset of mental disorder more than 2 years prior to the survey. We found that people with high levels of non-specific psychological distress were more likely to be current smokers. They were as likely as people with low levels of psychological distress to report wanting to quit smoking, trying to quit smoking, and to have used smoking cessation aids. However, they were significantly less likely to have quit smoking.

**Conclusions:**

The strong association between K6 psychological distress scores and mental disorders of long duration suggests that the K6 measure is a useful proxy for ongoing mental health problems. As people with anxiety and depressive disorders make up a large proportion of adult smokers in the US, attention to the role of these disorders in smoking behaviours may be a useful area of further investigation for tobacco control.

## Background

The health consequences of smoking have been long known and are well described [1,2]. As smoking rates in countries such as the US and Australia have declined substantially from their peak in the 1950s and 1960s it has become clear that there is large individual variation in level of addiction to nicotine, with some finding quitting smoking easier than others [3-5]. Further, evidence suggests that some people respond more strongly than others to population-based tobacco control initiatives including restrictions on marketing and sale of tobacco products, advertising of the health effects of smoking, the stigmatising of smoking behaviours, tobacco tax increases and price controls on tobacco [6,7]. A range of socio-demographic characteristics, including ethnicity, educational attainment, marital status and income have previously been identified that are associated with different rates of smoking initiation and smoking cessation [8,9]. However, studies of socio-demographic correlates of smoking and smoking cessation rarely consider the role of mental illness and its relationship to both smoking and socio-economic status.

There is a strong association between smoking and mental disorders, and around one-third of adult smokers in the US and Australia have been found to have DSM-IV and ICD-10 mental disorders. In comparison only 15% of adults who are not current smokers have diagnosable mental disorders [10,11]. While there seems general agreement that smoking is more prevalent among people with mental disorders, there is disagreement as to whether people with mental health problems are less likely than those without mental health problems to want to quit smoking or to be able to quit smoking [12,13]. This is of significance for tobacco control policy as there is large public expenditure on a range of measures designed to promote smoking cessation in adults. These measures have rarely been specifically designed for or targeted at people with mental illness in the belief that population-wide strategies are effective in promoting smoking cessation across the whole population [14-16].

Previous studies have investigated the relationship between smoking cessation attempts and outcomes, but these have mainly focused on socio-demographic predictors of quit attempts and successful smoking cessation [8,9,17-19]. Where mental disorders have been associated with less successful smoking cessation [13], it is unclear if this is related to reduced motivation to quit smoking resulting in less frequent smoking cessation attempts. Previous studies have found that higher levels of motivation and readiness to quit smoking were associated with successful quitting, but that higher levels of nicotine dependence were more strongly associated with failure to quit smoking [8,20]. As mental illness is associated with higher levels of nicotine dependence it is possible that mental illness could be a factor in failing to quit smoking [21,22]. Nicotine is a psychostimulant that affects several neuroregulators that influence behaviour and mood [23,24]. In some circumstances, nicotine can relieve symptoms of both depression and anxiety [25,26]. These factors have lead to the self-medication or mood maintenance hypothesis - that smokers with mental illness choose to smoke because it is the easiest, most readily accessible way to control symptoms of mental illness, especially for those who are not receiving any prescribed form of treatment for their mental health condition [27].

In consideration of the high proportion of US adult smokers who have common mental disorders, we set out to add to previous research by investigating the relationship between mental health, smoking cessation attempts and successful smoking cessation. The aims of the study were to test the following hypotheses:

a) rates of smoking increase with increasing level of non-specific psychological distress after adjusting for socio-demographic factors including education, income and marital status

b) among adults who smoke, rates of wanting to quit smoking, attempting to quit smoking and using smoking cessation aids do not vary by level of non-specific psychological distress

c) among adults who smoke, rates of quitting smoking decrease with increasing level of non-specific psychological distress.

To test these hypotheses we used data from the 2005 US National Health Interview Survey (NHIS), a large representative sample of the US population (n = 31,428). The NHIS uses the K6 scale to assess non-specific psychological distress [28]. This scale has been shown to be strongly associated with current diagnoses of mental health conditions [29]. However the scale specifically asks respondents to rate their level of psychological distress in the 30 days prior to the survey. It is possible that the scale would identify both short-term, transient, and long-standing psychological distress. We are unaware of any previous work which has examined the duration of mental disorders identified by the K6 scale. As some have argued that common mental disorders such as anxiety and depressive disorders are unlikely to influence smoking cessation behaviours as these mental disorders are likely to be transient while smoking is a long-term behaviour [12], we also set out to investigate the relationship between duration of mental disorder and reported level of psychological distress on the K6 scale. To do this, we also examined data from the Australian National Survey of Mental Health and Wellbeing (NSMHWB). This study was a large representative probability sample of adults in Australia (n = 8,841) which administered the K6 scale, along with a detailed structured assessment of mental disorders according to ICD-10 and DSM-IV criteria, including an evaluation of onset of symptoms. These data allowed us to examine the relationship between current levels of psychological distress and onset and duration of mental disorders.

## Methods

### Study population

We used data from the 2005 United States National Health Interview Survey (NHIS) [30]. The NHIS is a large, population-based representative survey of the US civilian non-institutionalised population, which has been run annually since 1957. In addition to core questions included every year, regular supplements are added to the survey. The cancer control supplement, which includes more detailed information about smoking behaviours, is run 5-yearly with the most recent data available from the 2005 survey. The NHIS uses a multi-stage area-based probability sampling design. The survey has three main components: the family component, the sample child and the sample adult. The family component contains information on household composition, demographic characteristics and basic health indicators. For each selected family, one child and one adult are selected to complete more detailed questionnaires. The cancer control supplement is administered to the selected adult participant. The survey questionnaires are administered by means of computer-assisted personal interviewing. The latest redesign of the NHIS was introduced in 1997, and included a measure of non-specific psychological distress, the K6 scale, which was developed for the survey [28].

As the study consisted of analysis of publicly available confidentialised files, no institutional ethics approval was required.

### Cohort identification

We used the full sample of 31,428 NHIS respondents to examine the relationship between socio-demographic characteristics, psychological distress and current smoking. We used the subset of 6,511 current smokers in the NHIS to examine the relationship between psychological distress and wanting to quit smoking, trying to quit smoking and using smoking cessation aids.

**Figure 1:**
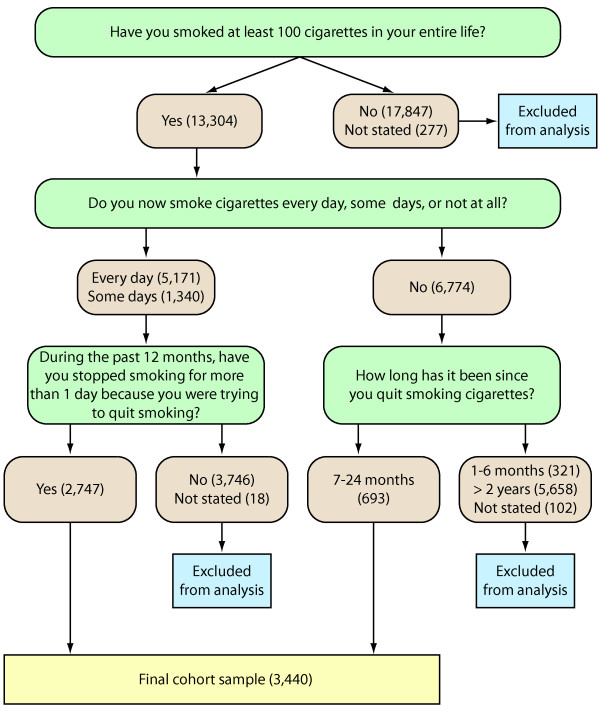
**Screening process used to select former smokers who quit within the last two years and current smokers with a recent failed quit attempt, US National Health Interview Survey**.

To examine the relationship between outcome of smoking cessation attempts, psychological distress and socio-demographic characteristics, we selected a cohort comprising recent successful quitters (those who reported having quit smoking 7-24 months prior to the survey, and who had not relapsed in that time) with current smokers who had unsuccessfully attempted to quit smoking in the past 12 months. Respondents were asked "Have you smoked at least 100 cigarettes in your entire life?", and if yes, "Do you now smoke cigarettes every day, some days, or not at all?" Respondents who said they smoked every day or some days were asked "During the past 12 months, have you stopped smoking for more than 1 day because you were trying to quit smoking?" Those that said yes were identified as the group of *current smokers with a recent unsuccessful quit attempt*. Respondents who don't currently smoke were asked "How long has it been since you quit smoking cigarettes?" Those who responded by choosing the category 7-24 months were identified as the group comprising *recent successful quitters*. The screening process used to define these two groups is illustrated in Figure 1. Because of the categories used in the NHIS the reference time periods for the two categories are slightly different, with current smokers with a recent unsuccessful quit attempt having attempted to quit smoking in the last 12 months, while recent successful quitters had quit smoking 7-24 months prior to the survey. A longer duration is required to define successful smoking cessation than relapse to smoking after a quit attempt.

### Independent variables

A range of demographic characteristics collected in the NHIS was used. Age was grouped in 10 year groups up to 65 years and over (with the youngest group coded as 18-24 years). Educational status was divided into less than high school graduate, high school graduate or GED (generalised equivalency diploma), some college or associate degree, and college graduate or higher. Family income is considered a sensitive data item, and not all respondents are willing to report their income. Where this occurs, respondents are asked to indicate if their family income is above or below $20,000 per annum. For respondents who provided their income, we grouped family income into four categories: under $20,000, $20,000-$44,999, $45,000-$74,999, $75,000 and above. Respondents who didn't provide their full income were separately classified into above or below $20,000. We also used sex, marital status, whether born in the United States, and housing tenure.

#### Mental health

Non-specific psychological distress was measured in the NHIS using the K6 non-specific psychological distress scale [28,31]. The K6 asks respondents about the 30 days prior to the survey. As many mental health problems can be persistent, we sought to investigate the relationship between the K6 measure of non-specific psychological distress and the diagnosis of ICD-10 mental disorders using data from another source - the Australian National Survey of Mental Health and Wellbeing (NSMHWB), which was conducted in 2007 [32,33]. The NSMWHB used a similar collection and field methodology to the US NHIS. The NSMWHB administered both the K6 scale, and the Composite International Diagnostic Interview (CIDI) [34], a detailed structured questionnaire that applies ICD-10 and DSM-IV criteria to diagnose mental disorders. It also collects information about onset and recency of disorders. We used data from the NSMHWB to estimate, in Australian adults, the proportion of people in each level on the K6 scale who were identified as having either an anxiety disorder (panic disorder, social phobia, agoraphobia, generalised anxiety disorder, post-traumatic stress disorder or obsessive-compulsive disorder) or a depressive disorder (depressive episode, dysthymia or bipolar affective disorder) during the last 12 months using the CIDI. Where a CIDI diagnosed disorder was identified, we used information collected on age of onset of that disorder.

### Statistical analyses

Data from both the US NHIS and the Australian NSMHWB have been weighted to account for the sample design and response patterns to allow estimation for the US or Australian adult population. All analysis was conducted using SAS software, Version 9.2 [35]. We used SAS Survey procedures (SURVEYFREQ, SURVEYLOGISTIC) to account for the clustered sample design and weighting. In fitting logistic regression models, all models were checked including testing for multicollinearity, checking residuals and testing for observations with high influence on the regression. Because of differences in the information provided on the public use microdata files, we used the method of linearisation in Taylor series to calculate design-adjusted standard errors and confidence intervals from the US NHIS, and jackknife replicate weights to calculate standard errors and confidence intervals from the Australian NSMHWB [36]. We used the Rao-Scott chi-square test to account for the survey design when testing for independence in two-way tables [37].

## Results

### Non-specific psychological distress and duration of anxiety and depressive disorders

Because the K6 scale specifically refers to the 30 days prior to the survey, we first examined data from the Australian NSMHWB to test the relationship between the K6 measure of non-specific psychological distress, ICD-10 mental disorders as identified using the CIDI, and first onset of anxiety or depressive disorders. We found that the distribution of K6 scores in Australia was similar to that found in the US NHIS, particularly in the higher levels of distress categories (Table 1). In Australian adults there was a strong association between high K6 scores and ICD-10 diagnosis of anxiety or depressive disorders (Table 2).

**Table 1:** Population distribution by level of non-specific psychological distress (K6), US National Health Interview Survey (n = 31,428) and Australian National Survey of Mental Health and Wellbeing (n = 8,841)

	Proportion of US civilian non-institutionalised adult population (NHIS)		Proportion of Australian adult population (NSMHWB)	
	%	95% CI	%	95% CI
K6 score				
0 - 2	69.3	(68.7 - 69.9)	62.3	(60.9 - 63.8)
3 - 7	19.8	(19.2 - 20.3)	29.1	(27.6 - 30.6)
8 - 12	6.2	(5.9 - 6.5)	6.2	(5.6 - 6.8)
13 - 24	2.9	(2.7 - 3.1)	2.3	(1.9 - 2.8)
Not stated	1.8	(1.6 - 2.0)	0.0	(0.0 - 0.1)

**Table 2:** Proportion of Australian adults with anxiety or depressive disorders, by level of non-specific psychological distress, Australian National Survey of Mental Health and Wellbeing (n = 8,841)

	%	95% CI
K6 level of non-specific psychological distress		
0 - 2	7.1	(6.1 - 8.1)
3 - 7	25.3	(23.1 - 27.5)
8 - 12	52.9	(46.9 - 58.9)
13 - 24	80.0	(73.7 - 86.3)

Note: Diagnosis of any anxiety or depressive disorder in the past 12 months using the CIDI.

**Figure 2:**
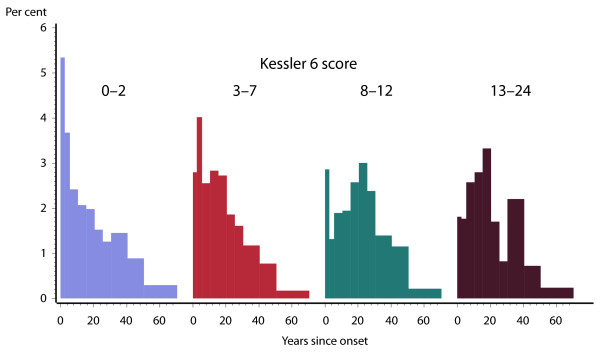
**Australian Adults with ICD-10 depressive or anxiety disorders - time since onset of symptoms by level of non-specific psychological distress, Australian National Survey of Mental Health and Wellbeing**.

For Australian adults who were diagnosed with depressive or anxiety disorders in the NSMHWB, the survey ascertained date of first onset of symptoms. For those adults with high or very high K6 scores who were given an ICD-10 diagnosis of an anxiety or depressive disorder, the vast majority had first onset of disorder more than 10 years prior to the survey. Less than 10% reported onset within two years of the survey. Even though the K6 questions ask about the 30 days prior to the survey, the vast majority of people with disorders reporting high levels of psychological distress have had their disorder for many years. Among people with K6 scores in the range 13-24, 90.2% (95% CI: 84.5%-95.8%) had first onset of symptoms more than 5 years prior to the survey. Among individuals with K6 scores in the range 8-12, 88.9% (95% CI: 84.1%-93.7%) had first onset more than 5 years prior to the survey, and among those with K6 scores in the range 3-7, 80.9% (95% CI: 77.5%-84.4%) had first onset more than 5 years prior to the survey (Figure 2).

### Association between smoking, psychological distress and socio-demographic factors in the 2005 NHIS

Table 3 shows the estimated population distribution and smoking rates by various demographic and psychosocial factors from the NHIS. The rate of smoking was lower in people aged 55 years and over, and was higher in men than women. Smoking rates were lower in people with college degrees, in people with higher incomes, people who own or are purchasing their own homes, and people not born in the United States. People with high levels of psychological distress have much higher smoking rates. A multivariate logistic regression model was fitted to these data estimating the odds ratios for current smoking. These results show that rate of smoking increased with level of non-specific psychological distress, and people with high levels of psychological distress were around twice as likely to be current smokers after adjusting for other socio-demographic factors.

**Table 3:** Proportion of population, proportion who smoke, and odds ratios for current smoking, by demographic and psychosocial characteristics, US National Health Interview Survey (n = 31,428)

	Frequency		Proportion who smoke		Multivariate odds ratio for current smoking	
						
	%	95% CI	%	95% CI	OR	95% CI
Age group (years)						
18 - 24	13.0	(12.5 - 13.5)	24.4	(22.4 - 26.3)	1.00	(Reference)
25 - 34	17.9	(17.4 - 18.4)	24.6	(23.2 - 25.9)	1.32	(1.18 - 1.47)
35 - 44	19.7	(19.2 - 20.3)	23.6	(22.4 - 24.8)	1.30	(1.16 - 1.46)
45 - 54	19.3	(18.8 - 19.9)	24.4	(23.1 - 25.7)	1.29	(1.15 - 1.46)
55 - 64	13.9	(13.4 - 14.3)	18.5	(17.2 - 19.8)	0.86	(0.76 - 0.98)
65 and over	16.1	(15.7 - 16.5)	8.6	(7.8 - 9.4)	0.27	(0.24 - 0.31)
Sex						
Male	48.2	(47.5 - 48.8)	23.9	(23.0 - 24.7)	1.63	(1.54 - 1.73)
Female	51.8	(51.2 - 52.5)	18.1	(17.5 - 18.8)	1.00	(Reference)
Race/ethnicity						
Non-Hispanic White	71.3	(70.8 - 71.9)	22.0	(21.3 - 22.6)	1.00	(Reference)
Hispanic	12.8	(12.4 - 13.1)	16.2	(15.0 - 17.4)	0.54	(0.49 - 0.60)
Non-Hispanic Black	11.3	(10.9 - 11.7)	21.3	(19.8 - 22.8)	0.64	(0.59 - 0.70)
All other race groups	4.6	(4.3 - 4.9)	16.4	(13.9 - 18.9)	0.89	(0.74 - 1.06)
Marital status						
Married/living with partner	61.8	(61.2 - 62.5)	18.9	(18.3 - 19.6)	1.00	(Reference)
Divorced/widowed/separated	17.0	(16.6 - 17.4)	24.0	(22.9 - 25.1)	1.41	(1.30 - 1.52)
Never married	19.7	(19.2 - 20.3)	24.3	(22.9 - 25.8)	1.02	(0.94 - 1.11)
Not stated	1.4	(1.3 - 1.6)	22.4	(18.5 - 26.4)	1.20	(0.97 - 1.48)
Education						
Less than high school	16.1	(15.6 - 16.6)	26.5	(24.9 - 28.0)	3.39	(3.03 - 3.78)
High school graduate or GED	29.4	(28.8 - 30.0)	26.8	(25.7 - 27.9)	2.99	(2.73 - 3.29)
Some college/associate degree	28.0	(27.4 - 28.5)	22.0	(21.0 - 23.0)	2.21	(2.01 - 2.43)
College graduate or higher	25.5	(24.9 - 26.0)	9.6	(8.8 - 10.3)	1.00	(Reference)
Not stated	1.1	(1.0 - 1.3)	19.3	(13.9 - 24.6)	2.69	(1.92 - 3.77)
Born in the United States						
Yes	84.2	(83.8 - 84.7)	22.2	(21.6 - 22.8)	1.00	(Reference)
No	15.7	(15.2 - 16.1)	14.1	(12.9 - 15.2)	0.52	(0.47 - 0.58)
Not stated	0.1	(0 - 0.1)	28.0	(7.6 - 48.3)	1.55	(0.58 - 4.19)
Family income						
Under $20,000	17.3	(16.8 - 17.7)	26.9	(25.7 - 28.0)	1.55	(1.38 - 1.75)
$20,000 or more not further stated	13.4	(13.0 - 13.9)	16.3	(14.8 - 17.7)	1.14	(1.01 - 1.30)
$20,000 - $44,999	22.1	(21.5 - 22.6)	25.2	(24.0 - 26.4)	1.52	(1.36 - 1.68)
$45,000 - $74,999	18.1	(17.6 - 18.6)	21.4	(20.0 - 22.7)	1.30	(1.17 - 1.45)
$75,000 or more	23.1	(22.5 - 23.6)	14.8	(13.7 - 15.9)	1.00	(Reference)
Not stated	6.1	(5.8 - 6.4)	20.6	(17.9 - 23.2)	1.34	(1.15 - 1.56)
Housing tenure						
Owned or being bought	70.9	(70.3 - 71.5)	17.9	(17.3 - 18.5)	1.00	(Reference)
Rented	26.4	(25.9 - 27.0)	28.6	(27.6 - 29.7)	1.52	(1.41 - 1.63)
Other	2.1	(1.9 - 2.3)	26.6	(22.9 - 30.2)	1.18	(0.99 - 1.41)
Not stated	0.6	(0.5 - 0.7)	16.6	(10.4 - 22.8)	0.73	(0.47 - 1.15)
K6 level of psychological distress						
0 - 2	69.3	(68.7 - 69.9)	17.7	(17.1 - 18.3)	1.00	(Reference)
3 - 7	19.8	(19.2 - 20.3)	25.2	(23.9 - 26.5)	1.41	(1.31 - 1.52)
8 - 12	6.2	(5.9 - 6.5)	33.9	(31.4 - 36.3)	1.89	(1.70 - 2.10)
13 - 24	2.9	(2.7 - 3.1)	41.9	(38.4 - 45.4)	2.41	(2.10 - 2.76)
Not stated	1.8	(1.6 - 2)	16.4	(12.5 - 20.4)	0.91	(0.70 - 1.18)

### Desire to quit and quit attempts by level of non-specific psychological distress

The NHIS asked current smokers if they a) would like to completely quit smoking cigarettes, b) if they had stopped smoking for more than one day in the past 12 months because they were trying to quit smoking, and c) whether they had used nicotine gum, patches, spray, inhalers, lozenges or prescription pills to try to quit smoking. The proportion of people wanting to quit smoking was lowest among people with K6 scores in the lowest range (0-2) at 61.6%. The proportion of people wanting to quit smoking was significantly higher in each other category of K6 score (p < 0.0001, p < 0.0001 and p = 0.044, respectively). The apparent drop in proportion wanting to quit smoking between people with K6 scores in the 8-12 range and people with K6 scores 13 and over was not statistically significant (p = 0.100) (Table 4). Similarly the proportion of people who had tried to quit smoking in the past 12 months was lowest among people with K6 scores in the lowest range at 37.8%. The proportion of people having tried to quit was significantly higher in each other category of K6 score (p < 0.001, p < 0.001, p = 0.003, respectively). The apparent drop in proportion trying to quit smoking between people with K6 scores in the 8-12 range and people with K6 scores 13 and over was not statistically significant (p = 0.082 (Table 4). There was no difference in rate of smokers who have tried to quit smoking using smoking cessation aids by level of non-specific psychological distress (Rao-Scott Χ² = 4.05, p = 0.67) (Table 5). People with high levels of psychological distress wanted to quit smoking, tried to quit smoking and used aids to quit smoking at least as frequently as anyone else.

**Table 4:** Current smokers: Whether wanted to quit smoking and whether tried to quit smoking in past year, by level of non-specific psychological distress, US National Health Interview Survey (n = 6,511)

	Want to quit smoking		Tried to quit smoking	
				
	%	95% CI	%	95% CI
K6 level of non-specific psychological distress				
0 - 2	61.6	(59.7 - 63.4)	37.8	(36.0 - 39.7)
3 - 7	69.5	(66.7 - 72.3)	48.8	(45.8 - 51.8)
8 - 12	73.0	(69.2 - 76.9)	52.8	(48.4 - 57.3)
13 - 24	67.5	(62.0 - 72.9)	46.6	(41.1 - 52.0)

**Table 5:** Current smokers who have tried to quit smoking in the past year: Whether used aids (nicotine gum, patch, spray, inhaler, lozenge, or prescription pill), by level of non-specific psychological distress, US National Health Interview Survey (n = 2,747)

	**%**	**95% CI**
K6 level of non-specific psychological distress		
0 - 2	27.3	(24.6 - 30.1)
3 - 7	30.6	(26.6 - 34.5)
8 - 12	30.1	(24.4 - 35.8)
13 - 24	32.2	(25.1 - 39.3)

### Comparison of recent successful quitters and current smokers who have attempted to quit

Figure 1 shows the selection of recent quitters and current smokers who have tried to quit from the 2005 NHIS. The cohort sample of 3,440 adults comprised 2,747 current smokers who have tried quitting in the past 12 months, and 693 former smokers who quit 7-24 months prior to the survey. Demographic and psychosocial characteristics of the two groups are shown in Table 6. The table also shows the results of a multivariate logistic regression model fitted to these data predicting odds of successfully quitting smoking accounting for the complex sample design. Low education and single marital status were associated with a reduced likelihood of successfully quitting smoking. Higher levels of psychological distress were also associated with reduced odds of quitting smoking, after adjusting for socio-economic factors. Housing tenure, place of birth and race were not found to be associated with successful quitting and were eliminated from the final model.

**Table 6:** Selected characteristics of US adults, by smoking cessation status, and odds ratios comparing those who successfully quit smoking with unsuccessful quitters, US National Health Interview Survey

	Current smokers who attempted but failed to quit in past year (n = 2,747)		Former smokers who quit 7-24 months ago (n = 693)		Multivariate odds ratio for successful quitting	
						
	%	95% CI	%	95% CI	OR	95% CI
Age group (years)						
18 - 24	17.3	(15.4 - 19.2)	16.4	(12.6 - 20.1)	1.00	(Reference)
25 - 34	23.2	(21.3 - 25.0)	26.7	(22.8 - 30.6)	0.97	(0.68 - 1.39)
35 - 44	22.6	(20.8 - 24.4)	18.0	(14.9 - 21.1)	0.66	(0.45 - 0.97)
45 - 54	20.7	(18.9 - 22.6)	16.3	(13.1 - 19.5)	0.63	(0.42 - 0.95)
55 - 64	10.9	(9.6 - 12.1)	13.0	(10.3 - 15.7)	0.99	(0.65 - 1.50)
65 and over	5.4	(4.5 - 6.3)	9.7	(7.2 - 12.1)	1.68	(1.05 - 2.68)
Sex						
Male	52.7	(50.5 - 54.9)	56.6	(52.3 - 60.8)	1.17	(0.96 - 1.43)
Female	47.3	(45.1 - 49.5)	43.4	(39.2 - 47.7)	1.00	(Reference)
Race/ethnicity						
Hispanic	9.6	(8.5 - 10.8)	11.1	(8.7 - 13.6)	(a)	
Non-Hispanic White	73.2	(71.3 - 75.1)	75.1	(71.4 - 78.8)		
Non-Hispanic Black	13.2	(11.8 - 14.6)	10.4	(7.7 - 13.2)		
All other race groups	4.0	(3.0 - 4.9)	3.4	(1.7 - 5.0)		
Marital status						
Married/living with partner	55.1	(52.9 - 57.3)	62.4	(58.3 - 66.6)	1.00	(Reference)
Divorced/widowed/separated	18.9	(17.4 - 20.4)	16.0	(13.2 - 18.7)	0.79	(0.61 - 1.03)
Never married	24.7	(22.7 - 26.7)	20.5	(16.8 - 24.2)	0.72	(0.53 - 0.96)
Not stated	1.2	(0.8 - 1.6)	1.1	(0.4 - 1.9)	0.79	(0.34 - 1.82)
Education						
Less than high school	18.5	(16.8 - 20.3)	16.2	(13.2 - 19.2)	0.57	(0.41 - 0.80)
High school graduate or GED	35.0	(32.8 - 37.1)	31.4	(27.2 - 35.6)	0.56	(0.41 - 0.76)
Some college/associate degree	33.5	(31.4 - 35.5)	31.0	(27.0 - 35.0)	0.59	(0.44 - 0.79)
College graduate or higher	12.4	(11.0 - 13.9)	20.6	(17.1 - 24.1)	1.00	(Reference)
Not stated	0.6	(0.3 - 0.9)	0.8	(0.0 - 1.7)	0.87	(0.28 - 2.66)
Born in the United States						
Yes	10.1	(8.8 - 11.4)	10.5	(8.1 - 13.0)	(a)	
No	89.8	(88.5 - 91.2)	89.5	(87.0 - 91.9)		
Not stated	0.1	(0.0 - 0.2)	0.0	(0.0 - 0.2)		
Family income						
Under $20,000	22.0	(20.4 - 23.6)	15.9	(13.3 - 18.6)	0.77	(0.55 - 1.09)
$20,000 or more not further stated	9.1	(7.7 - 10.6)	11.8	(8.8 - 14.8)	1.11	(0.74 - 1.67)
$20,000 - $44,999	26.2	(24.3 - 28.1)	25.5	(21.7 - 29.3)	0.86	(0.62 - 1.20)
$45,000 - $74,999	19.9	(18.0 - 21.7)	20.7	(17.1 - 24.2)	0.86	(0.62 - 1.19)
$75,000 or more	17.8	(15.9 - 19.7)	21.6	(17.8 - 25.4)	1.00	(Reference)
Not stated	5.0	(4.1 - 5.9)	4.5	(2.6 - 6.3)	0.75	(0.44 - 1.27)
Housing tenure						
Owned or being bought	60.1	(58.0 - 62.2)	64.5	(60.4 - 68.6)	(a)	
Rented	36.7	(34.7 - 38.8)	33.2	(29.1 - 37.2)		
Other	2.8	(2.1 - 3.5)	2.1	(0.9 - 3.2)		
Not stated	0.4	(0.1 - 0.7)	0.3	(0 - 0.7)		
K6 level of psychological distress						
0 - 2	52.5	(50.3 - 54.7)	66.8	(62.8 - 70.8)	1.00	(Reference)
3 - 7	27.5	(25.5 - 29.6)	22.2	(18.6 - 25.7)	0.79	(0.64 - 0.97)
8 - 12	12.6	(11.1 - 14.1)	5.9	(4.1 - 7.7)	0.52	(0.38 - 0.72)
13 - 24	6.5	(5.5 - 7.5)	4.5	(2.8 - 6.1)	0.62	(0.42 - 0.92)
Not stated	0.9	(0.5 - 1.3)	0.6	(0.0 - 1.3)	0.56	(0.19 - 1.63)

(a) Variable not significantly associated with successful quitting status, and eliminated from final model

## Discussion

### Relationship between psychological distress, mental disorders and smoking

The K6 scale is a useful short measure for assessing mental health problems in omnibus surveys. It was specifically designed for surveys such as the NHIS which seek to assess a large number of topics, and thus need effective, short screening scales. Our results showing that the K6 score was strongly associated with ICD-10 diagnosis of anxiety or depressive disorders are consistent with previous findings [29,32]. Although the K6 questions ask about the 30 days prior to the survey, we found that most people with high levels of non-specific psychological distress have long-standing mental disorders. This suggests that high K6 scores are unlikely to be identifying short-term or transient mental health conditions. As far as we know, these are the first data showing that high levels of non-specific psychological distress are associated with mental disorders of long duration.

The K6 has an additional advantage of being a dimensional scale, with higher scores being associated with higher levels of psychological distress. It has been suggested that research based on applying ICD-10 or DSM-IV diagnostic criteria may underestimate the contribution of mental illness to smoking behaviours by ignoring the contribution of sub-threshold disorders [38]. Studies that use ICD-10 or DSM-IV diagnoses via structured interviews have estimated that one third of adult smokers in the US have mental disorders [10,11]. Our results show an increasing trend of smoking rates and decreasing rates of smoking cessation even for moderate levels of psychological distress, consistent with the idea that mental disorders of mild and moderate severity are significant risk factors for smoking and inhibitors of smoking cessation [13]. Thus the contribution of mental illness to smoking initiation, delayed smoking cessation and longer duration of smoking may be substantial.

The relationship between smoking and severe mental illness is well known [39,40]. Previous research using the K6 scale [41-43] has used the highest band of the scale, scores of 13 and above, to define the category of serious psychological distress, which is highly correlated with severe mental disorder requiring clinical care [28,29]. Approximately 3% of the US population falls in this range, according to the NHIS data. Studies using structured diagnostic instruments, such as the CIDI, estimate a prevalence of 12-month mental disorders of around 20% in the US and Australia [32,44]. These studies include more common disorders such as anxiety and depressive disorders that are less severe than the definition of severe mental illness or serious psychological distress. Nevertheless, smoking rates have been found to be high using this broader definition of mental disorders, and even people with anxiety or depressive disorders of mild or moderate severity have higher rates of smoking initiation and lower rates of smoking cessation [10,11,13]. Some have argued that the relatively high prevalence rates of mental disorders estimated in epidemiological surveys are unrealistic as specialist clinical services could not be provided to 20% of the population, and that the definition of mental disorders should be limited to those disorders severe enough to require clinical intervention or justify receipt of disability benefits [12,45,46]. Physical health problems as a broad group are also highly prevalent, and common chronic conditions such as asthma, hypertension, diabetes and metabolic syndrome are burdensome and require management even if they do not require immediate hospitalisation. Similarly, the broader definition of mental disorders encompassed by ICD-10 and DSM-IV includes disorders that may not require immediate clinical treatment, but are nevertheless burdensome and impact on people's functioning and their ability to contribute to society. Smoking rates in this group are double that of people with no lifetime mental disorders [10,11]. Our results show that the approximately 20% of the US adult population who score in the 3-7 range on the K6, well below the 13 and above cut-off for defining serious psychological distress, were 40% more likely to be current smokers, were more likely to want to or to try to quit smoking, but were 20% less likely to successfully quit smoking than people with K6 scores in the low range 0-2. While some attention has been directed to addressing smoking among people with severe mental illness in contact with specialist services [47], one implication of these findings is the need for non-service based responses to address the larger population of smokers with common mental disorders.

### Psychological distress and smoking cessation

We found that people with moderate and high levels of non-specific psychological distress were more likely than people with low levels of psychological distress to want to quit smoking and to try to quit smoking. People with moderate and high levels of psychological distress were also just as likely as those with low levels of non-specific psychological distress to use smoking cessation aids. However, they were significantly less likely to have successfully quit smoking for six months or more. Mental illness has been associated with higher levels of nicotine dependence, and higher intensity of smoking [48,49]. It is possible that this could contribute to lower rates of smoking cessation in people with mental illness.

People with moderate and high levels of non-specific psychological distress may require a higher level of support, or more specifically targeted strategies to support their smoking cessation efforts. Analysis of tobacco industry marketing research and market segmentation studies has shown that historically the tobacco industry has used psychosocial and other personality traits to develop targeted promotional strategies that encourage uptake of tobacco use [10,50,51]. A number of different brand variants have been brought to market with specifically targeted promotional strategies that took advantage of industry knowledge of the association between smoking and psychosocial problems [50-54]. In addition to developing brands or campaigns targeting youth or women, for example, the tobacco industry targeted market segments defined by psychosocial characteristics such as personality type. Campaigns and promotional strategies were developed around the use of tobacco products to address psychosocial issues such as reducing stress, anxiety or nervousness, improving mood, increasing confidence in social situations, or improving concentration [51,54]. In contrast, population-based strategies in tobacco control have much less often been targeted at specific demographics and other population groups [16]. The result is an inequality in outcomes from these population-based investments which seem to disadvantage smokers who are also dealing with mental health problems [11].

### Implications for health promotion and tobacco control

It is well recognised that the profile of adult smokers now is demographically and psychosocially very different from the profile of those who smoked a couple of decades ago [57]. That such a high proportion of current smokers in developed countries suffer from some form of mental illness or psychological distress has been recognised as representing a new and emerging challenge for tobacco control, as these smokers may perceive different benefits to smoking, and efforts to quit smoking may be complicated by their life circumstances and the interaction between psychiatric symptoms and neuroactive substances such as nicotine [55]. Nonetheless, outside of efforts to reduce smoking in psychiatric inpatient settings, policy documents describing directions in tobacco control do not propose any specific strategies for responding to these challenges or providing support to this large and vulnerable group [14,15,55].

It is known that many common mental disorders, in particular anxiety disorders, have onset during childhood and adolescence, and persist for many years [13]. Most adult smokers begin smoking and proceed to daily smoking in their teenage years [56]. While the causal pathways are not clearly understood and there is some evidence to support multiple pathways, the strong association between mental illness and smoking, and the common long-term persistence of both smoking and mental illness, suggest the value in responding to both problems in a co-ordinated way [57,58]. As a large proportion of smokers have common mental disorders, such efforts would likely be helpful to many people. There is some evidence that smoking cessation not only benefits long-term physical health, but may result in improvements in mental health as well [58]. In the short term, smoking cessation can result in exacerbation of anxiety or depressive symptoms, but in the longer term, smoking cessation is associated with an overall reduction in anxiety and stress [58].

Only a small proportion of people with high levels of non-specific psychological distress, and common mental disorders such as anxiety and depression, are treated by specialist mental health services [11]. The majority of people with these problems do not seek any professional help. Service-based responses to smoking and mental illness, such as smoke-free mental health units, and smoking cessation counselling as part of mental health treatments, will only reach a small proportion of the population of adult smokers with mental health problems. This suggests that targeted population-based strategies will be required. While the treatment of individuals with mental health problems has been moving to embrace a more holistic strategy of treating the whole person including any physical health problems and substance dependence issues they might have [59], population health has been slower to respond to this trend. Most population health initiatives are targeted at single diseases or risk factors, and are often coordinated by organisations that are focused on single issues or diseases. For instance, organisations that focus on heart disease, or cancer, which traditionally have had a strong interest in population-based tobacco control initiatives have been more likely to regard people with mental illness as the target group of some other organisation rather than a numerically significant, and vulnerable, part of their own target groups.

Changes in the socio-demographic profile of smokers over time, and the persistence of smoking despite long-term advertising of the health dangers have led to the development of the hardening hypothesis and identification of possible hard-core smokers [60]. This hypothesis is controversial [16], and is supported by only limited empirical data. It is underpinned by the concept of the hard-core smoker as someone who is stubbornly resistant to population tobacco-control initiatives. It is possible that some individuals who continue to smoke despite decades of publicity about the dangers of smoking, are facing other problems in their lives, such as mental health problems, that affect their ability to quit smoking. Better understanding of the characteristics and life circumstances of these heavily dependent smokers may help refocus tobacco control efforts in ways that help address the range of difficulties these people are facing.

### Limitations

Cross-sectional studies, such as the NHIS, describe associations but cannot inform causal pathways. It is not possible to conclude from these data whether psychological distress leads to smoking uptake or whether smoking causes psychological distress or whether both are related to some other causal factors. However, the data from the Australian NSMHWB showed that the vast majority of people who have moderate or high levels of non-specific psychological distress have mental disorders of long duration. It is reasonable to assume that a high proportion of people with moderate and high levels of non-specific psychological distress in the NHIS sample would have had long-standing elevated levels of psychological distress, predating quit attempts in the 12 months prior to the survey, in most cases by many years. A longitudinal study of Swedish male military conscripts found that lower levels of smoking cessation between ages 30 and 50 were predicted by higher levels of psychological distress measured at 18 years [61].

While the K6 scale has been shown to be a good predictor of mental disorders, and people with high K6 scores are likely to have mental disorders of long duration, K6 scores are not the same as making formal psychiatric diagnoses. Studies that use the K6 and the CIDI are primarily designed to assess mental disorders during discrete time intervals. It has also been suggested that the strong association between anxiety, depression and smoking could be conceptualised in terms of neuroticism - the general personality trait that encompasses long-term and persistent susceptibility to anxiety and depressive symptoms [62,63]. There is some evidence that short-term mental disorders in the absence of neurotic traits may have a weaker association with smoking behaviours [64,65]. While the K6 measure of psychological distress was found to be strongly associated with smoking status and inversely associated with successful smoking cessation, a more direct measure of long-standing mental disorders such as anxiety or depression may show even stronger associations.

## Conclusions

Overall, people with moderate or high levels of non-specific psychological distress are more likely to be current smokers. People with moderate or high levels of psychological distress are at least as likely as anyone else to report wanting to quit smoking, trying to quit smoking, and using smoking cessation aids. However, people with psychological distress are less likely to have successfully quit smoking. Common mental health problems such as anxiety and depression, which are highly correlated with psychological distress, are important, yet commonly overlooked factors, in tobacco control programs. As these mental health problems have high prevalence, and many people with these mental health problems do not receive treatment, both service-based and population-based initiatives will be required to address smoking in this population. To date, targeted population-based strategies in particular, seem to be an under-developed area. Attention to developing new programs and initiatives directed at smokers with common mental health problems may be helpful in reducing the high rates of smoking-related morbidity and mortality in this group.

## Competing interests

The authors declare that they have no competing interests.

## Authors' contributions

DL and FM conceived the original idea for the study. All authors contributed to the development of the study methodology. DL acquired and analysed the data, and wrote the first draft of the manuscript. All authors edited the paper. All authors read and approved the final manuscript.

## Pre-publication history

The pre-publication history for this paper can be accessed here:

http://www.biomedcentral.com/1471-2458/11/256/prepub
